# Risk factors for fistulizing disease of the pouch after ileal pouch-Anal anastomosis for ulcerative colitis

**DOI:** 10.1007/s00595-026-03246-8

**Published:** 2026-02-09

**Authors:** Yoshiki Okita, Yoshinaga Okugawa, Hiroki Imaoka, Tadanobu Shimura, Takahito Kitajima, Mikio Kawamura, Hiromi Yasuda, Yuhki Koike, Shigeyuki Yoshiyama, Masaki Ohi, Yuji Toiyama

**Affiliations:** 1 Departments of Gastrointestinal and Pediatric Surgery Division of Reparative Medicine Institute of Life Sciences, Mie University Graduate School of Medicine, 2-174 Edobashi, Mie 514-8507 Tsu, Japan; 2 Department of Genomic Medicine, Mie University Hospital, Mie Tsu, Japan

**Keywords:** Ulcerative colitis, Ileal pouch-anal anastomosis, Crohn’s disease of the pouch

## Abstract

**Purpose:**

Long-term data on the risk factors for fistulizing disease (FD) after ileal pouch-anal anastomosis (IPAA) for ulcerative colitis are limited. This study aimed to identify the predictors of FD after ileostomy closure and evaluate the impact of FD on pouch failure.

**Methods:**

We reviewed 374 patients who underwent mucosal restorative proctocolectomy with handsewn IPAA between 2004 and 2022. Cox proportional hazards regression and log-rank tests were used to assess the FD-free survival and risk factors.

**Results:**

After excluding 59 patients, 315 patients were analyzed. FD developed in 20 (6.3%) of these patients. Multivariate analysis showed that a monthly prednisolone (PSL) dose ≥ 450 mg before IPAA and chronic pouchitis were independent risk factors for FD (odds ratio [OR] 2.96, 95% CI: 1.15–7.64, *p* = 0.025; OR 3.85, 95% CI: 1.52–9.78, *p* = 0.0045). Patients with PSL ≥ 450 mg or chronic pouchitis had significantly poorer FD-free survival (*p* = 0.0004 and *p* = 0.0002, respectively). Pouch failure occurred more frequently in patients with FD than in those without FD (30.0% vs. 1.0%; *p* < 0.0001).

**Conclusions:**

High steroid exposure before IPAA and chronic pouchitis were significant predictors of FD, which strongly increased the risk of pouch failure.

**Supplementary Information:**

The online version contains supplementary material available at 10.1007/s00595-026-03246-8.

## Introduction

Restorative proctocolectomy and ileal pouch-anal anastomosis (IPAA) have become the definitive surgical procedures for ulcerative colitis (UC). Although the long-term pouch survival rate for UC is satisfactory [1–3], the outcome of this procedure may be compromised by pouch-related complications. Recent studies reported that Crohn’s disease (CD) of the pouch was one of the most frequent reasons for pouch failure. The presence of a fistula, stricture, or inflammation above the level of the pouch (prepouch ileitis) appears most consistent with the current definition of CD of the pouch [4]. Several hypotheses to explain the development of CD of the pouch have been suggested including misinterpretation of the original UC diagnosis before colectomy, de novo CD that develops after IPAA, or a new disease entity with Crohn’s-like features [5–7].

Fistulizing diseases (FD) of the pouch, such as pouch-perianal fistulas, pouch-vaginal fistulas, or presacral sinus, are other common causes of pouch failure [8, 9] and are generally considered long-term sequelae of untreated anastomotic leakage [10]. However, late-onset FD of the pouch develops in a small proportion of patients with UC, months to years after pouch creation and ileostomy closure, despite a well-healed pouch–anal anastomosis [10, 11]. Therefore, distinguishing between CD of the pouch and FD of the pouch, which is a possible postoperative complication after IPAA, is challenging because the phenotypes overlap [12].

The long-term follow-up data on the association between clinical factors and FD in UC patients with IPAA are inadequate. We conducted this study to identify the risk factors for the development of FD of the pouch after ileostomy closure following IPAA, and to evaluate the long-term influence of FD of the pouch on pouch failure.

## Materials and methods

### Study population

We reviewed the surgical UC database of Mie University retrospectively. This study was approved by the Institutional Review Board of Mie University (H2024-175). Informed consent was obtained from all patients for the use of their data.

## Standard procedure and timing of ileostomy closure

The standard operation for UC in our institute is mucosal restorative proctocolectomy below the dentate line with handsewn IPAA. The surgical technique was designed to reduce pouch fistula formation and was first performed in 2004 [13]. Therefore, only UC patients with IPAA who underwent this procedure from 2004 were evaluated. We reviewed the data for 374 patients who underwent IPAA between January, 2004 and December, 2022. All patients underwent the procedure as one-stage, two-stage (IPAA with diverting ileostomy and ileostomy closure), or three-stage (subtotal colectomy, IPAA with diverting ileostomy, and ileostomy closure) procedures using a 15–18-cm, two-limbed, J-shaped ileal pouch. We usually construct the ileal pouch by creating a small central opening, performing a side-to-side anastomosis, and then closing the opening with a stapler to complete the pouch. A gastrografin enema was administered routinely, to detect pouch leakage on postoperative days (PODs) 6 or 7 and just before ileostomy closure. The ileostomy was usually closed approximately 3–4 months after IPAA if an intact pouch without pouch leakage was confirmed.

The diagnosis of postoperative CD was based on a comprehensive evaluation of the pathological evaluation of the resected specimens, endoscopic findings, and clinical disease characteristics. We re-evaluated the resected specimens at the time of colectomy or proctectomy.

## Exclusion criteria

To identify the risk factors for FD of the pouch, the exclusion criteria were as follows: < 14 years of age, stapled IPAA, one-stage IPAA, pouch leakage before ileostomy closure or within 180 days after ileostomy closure, and postoperative diagnosis of CD after IPAA. Crohn’s disease was diagnosed in accordance with the preoperative Japanese diagnostic criteria and subsequently confirmed by pathological examination of the resected specimens, which revealed findings such as granulomas and skip lesions [14]. The cutoff of 180 days after ileostomy closure for pouch leakage was set to avoid confusion between pouch leakage and FD because pouch leakage typically occurs as an early complication before or within 180 days after ileotomy closure [15].

## Definitions of FD of the pouch and pouch failure

FD of the pouch was defined as pouch-vaginal fistula, pouch-perianal fistula, presacral sinus, or leakage from the pouch body or tip of the “J” more than 180 days after ileostomy closure. Pouch-body leakage originates either from the stapler line of the side-to-side anastomosis or from the stapler line used to close the central opening. FD of the pouch was usually diagnosed by pouchoscopy, gastrografin enema, and cross-sectional imaging (computed tomography or magnetic resonance imaging) to identify distinctive CD findings in the ileal pouch and proximal small intestine (Supplemental Table 1).

The follow-up period was defined as the time from ileostomy closure to the last outpatient visit or the date of diagnosis of FD of the pouch. Pouch failure was defined as a dysfunctional pouch requiring pouch excision or a permanent diversion.

## Definitions of the variables

The risk factors evaluated for FD of the pouch were as follows:

1. Patient-related factors: sex, age at UC onset, age at IPAA, year of IPAA (before or after June 2010, when infliximab was first covered by insurance in Japan and corresponds approximately to the introduction of laparoscopic surgery for UC in our institute), disease duration, colitis extent, disease type, colitis severity (Truelove and Witt’s criteria) just before IPAA (severe or not), cancer/dysplasia, history of pyoderma gangrenosum, and body mass index.
2. Medication-related factors: monthly dosage of prednisolone (PSL) just before IPAA, and the use of biologics, thiopurines, or calcineurin inhibitors.
3. Surgery-related factors: staged operation (two-stage or three-stage), approach (laparoscopy or open), intraoperative blood transfusion, surgical site infection, chronic pouchitis and preceding chronic pouchitis.

The monthly dosage of PSL before IPAA was calculated using the steroid dose prescribed just before IPAA, which was converted to PSL. Biologic therapy was defined as the administration of at least one infusion of infliximab within 8 weeks, adalimumab within 2 weeks, or ustekinumab within 8 weeks before IPAA. Thiopurine use was defined as at least one oral administration within 4 weeks before IPAA. Calcineurin inhibitor use was defined as at least one intravenous or oral administration of cyclosporine or tacrolimus within 2 weeks before IPAA.

Cutoff values for disease duration, body mass index, and steroid dosage per month just before IPAA were determined by receiver operating characteristic curve analyses for the occurrence of FD of the pouch.

### Definition of chronic pouchitis

The diagnosis of pouchitis was based on both clinical symptoms and endoscopic findings for all patients. Biopsies were not performed routinely. Pouchitis was defined as a modified Pouchitis Disease Activity Index score of ≥ 5 points and was further classified into acute and chronic pouchitis on the basis of antibiotic treatment efficacy in accordance with previous guidelines [16, 17]. Chronic pouchitis was defined as a diagnosis of chronic pouchitis before, or concomitantly with, the development of FD of the pouch. Chronic pouchitis was distinguished endoscopically from CD. Preceding chronic pouchitis was defined as chronic pouchitis identified concurrently with FD at the time of pouch endoscopy.

### Statistical analysis

Statistical analyses were performed using JMP Pro 16.2.0 (SAS Institute Inc, Cary, NC). All variables were fully recorded, with no missing data. Comparisons of the clinicopathological characteristics between groups were evaluated using Fisher’s exact test for categorical variables. Continuous variables are shown as medians with interquartile ranges and were compared using the Mann–Whitney U test. The FD of the pouch-free survival period was calculated from the date of ileostomy closure to the date of diagnosis of FD of the pouch, and patients were censored at the last follow-up date.

Survival analysis was performed using the Kaplan–Meier method and compared using the log-rank test. Cox proportional hazards models were used to estimate hazard ratios for the development of FD of the pouch. Factors associated with FD of the pouch with *p*-values < 0.05 in the univariate analysis were selected for the multivariate analysis using a Cox proportional hazards regression model; *p* < 0.05 was considered significant.

## Results

### Incidence of FD of the pouch after ileostomy closure

**Fig. 1:**
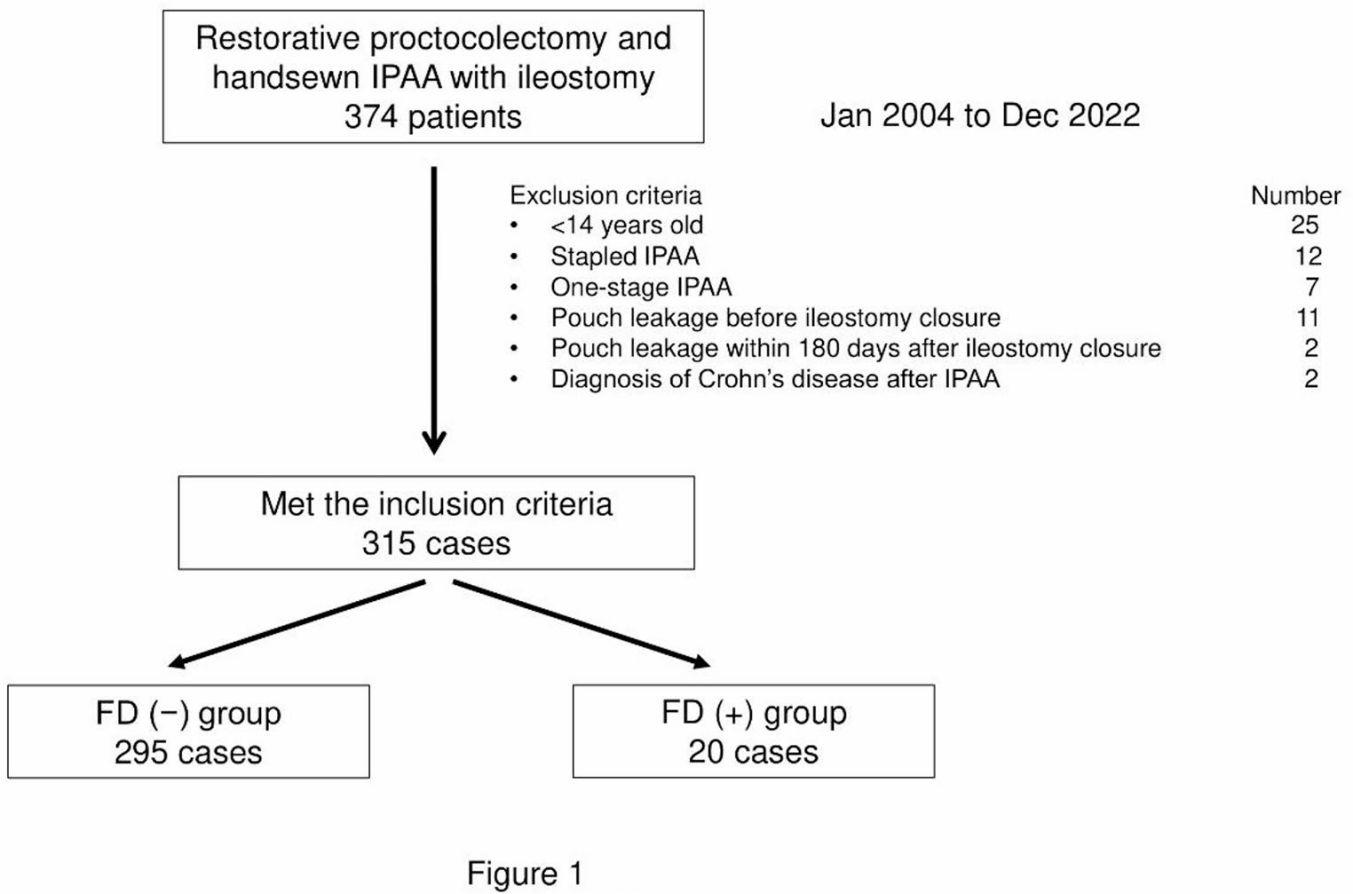
Flowchart of the comparison between patients with and those without fistulizing disease of the pouch. FD of the pouch developed in 20 (6.3%) of the 315 patients remaining after the exclusion of 59 patients who did not meet the criteria for analysis. IPAA = ileal pouch-anal anastomosis; FD = fistulizing disease

**Fig. 2:**
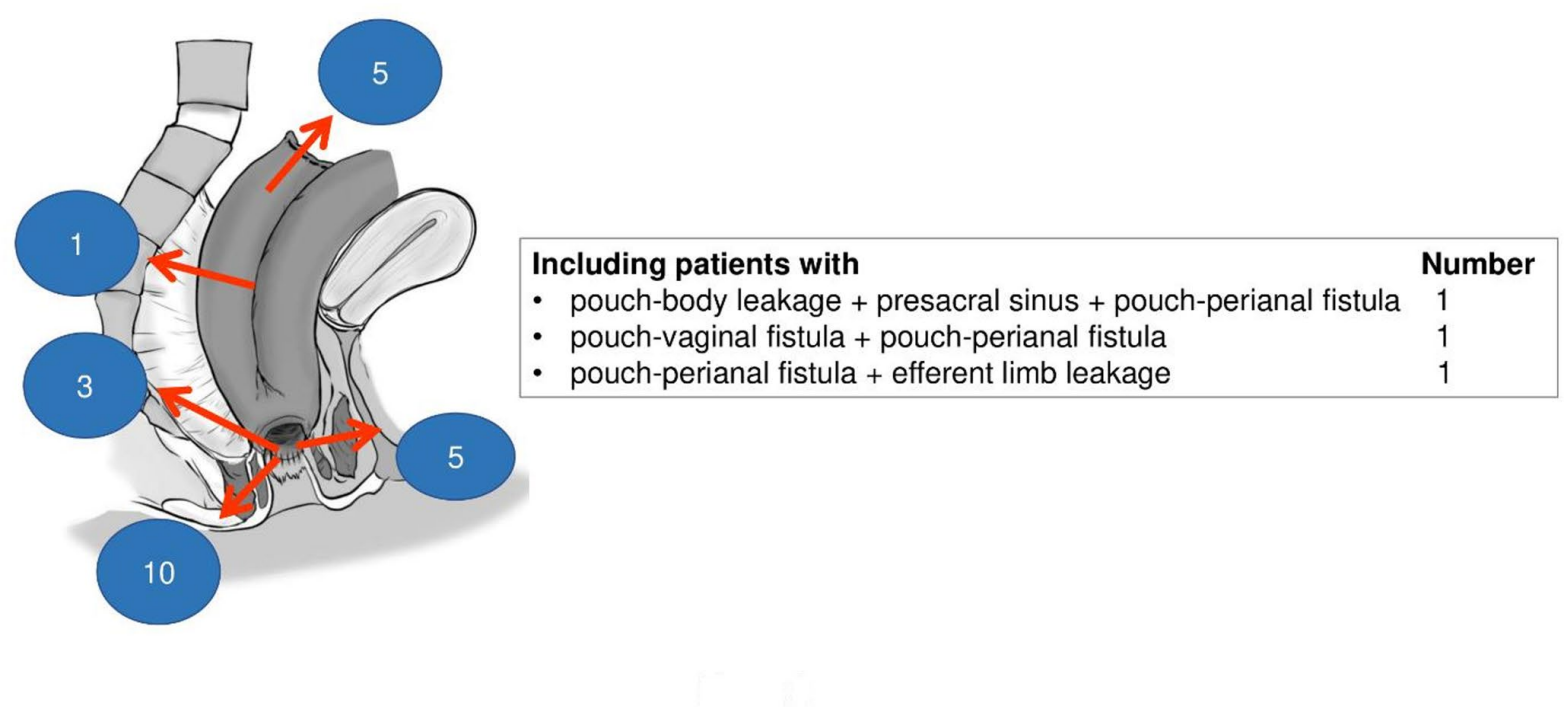
Exact causes of fistulizing disease (FD) of the pouch. Among the 20 patients with FD of the pouch, 10 had pouch-perianal fistulas, 5 had pouch-vaginal fistulas, 5 had efferent limb leakage, 3 had a presacral sinus, and 1 had pouch-body leakage. The numbers of patients with each type of fistula are shown in the blue circles. FD = fistulizing disease

After the exclusion of 59 patients based on the above criteria, 315 patients were included in the analysis. FD of the pouch was identified in 20 (6.3%) of these patients **(**Fig. 1**)**, with a mean follow-up period of 97.2 ± 59.9 months. The causes of FD of the pouch were pouch-perianal fistula in 10 patients, pouch-vaginal fistula in 5, efferent limb leakage in 5, presacral sinus in 3, and pouch-body leakage in 1. The patients with FD included one with pouch-body leakage, presacral sinus, and pouch-perianal fistula; one with both a pouch-vaginal fistula and a pouch-perianal fistula; and one with both a pouch-perianal fistula and efferent limb leakage (Fig. 2). Based on diagnostic modalities, including endoscopy, pouch–perianal fistulas (10 patients), pouch–vaginal fistulas (5 patients), and presacral sinuses (3 patients) were considered to originate from the ileoanal anastomosis. Efferent limb leakage without concomitant fistulizing disease (4 patients) was considered to originate from the suture line at the tip of the “J”. In one patient, pouch-body leakage, a presacral sinus, and a pouch–perianal fistula may also have originated from the ileoanal anastomosis. The duration from ileostomy closure after IPAA to the diagnosis of FD of the pouch was 72.2 ± 39.6 months.

The procedures used to identify FD of the pouch and exclude CD comprised pouchoscopy in 20 patients, computed tomography in 19, gastrografin enema in 19, and magnetic resonance imaging in 7. At the time of diagnosis of FD of the pouch, three patients had been taking oral prednisolone for steroid withdrawal syndrome, not for FD of the pouch. One patient received infliximab for chronic antibiotic refractory pouchitis. Among the patients diagnosed with FD of the pouch, 13 had chronic pouchitis, for which antibiotics were administered either continuously or episodically. The treatments for FD of the pouch are shown in Supplemental Table 1.

**Table 1:** Clinicopathological characteristics of patients with versus those without fistulizing disease of the pouch

Variable	Total *N*=315	FD(−) *N*=295	FD(+) *N*=20	*p*-value
Sex (Male/Female)	190/125	181/114	9/11	0.16
Age at ulcerative colitis onset (years)	29(21–43)	30(21–43)	24(19–32)	0.04
Age at IPAA (years)	39(28–52)	40(28–53)	30(24–33)	0.007
<18 years/>18 years	18/297	16/279	2/18	0.32
<65 years/≥65 years	17/298	17/278	0/20	0.61
Year at IPAA (before/after June 2010)	113/202	101/194	12/8	0.03
Disease duration (years)	5(1.5–11.5)	5(1.5–11.5)	3.5(1–8)	0.21
Extent of colitis: total colitis (Y/N)	274/41	257/38	17/3	0.73
Disease type: First attack (Y/N)	47/268	43/252	4/16	0.52
Severity of colitis: Severe (Y/N)	14/301	13/282	1/19	0.61
Concomitant cancer/dysplasia (Y/N)	43/272	42/253	1/19	0.33
History of pyoderma gangrenosum (Y/N)	8/307	7/288	1/19	0.41
Body mass index	20.5(17.5–23.2.5.2)	20.6(17.5–23.5.5.5)	19.0(17.7–21.1.7.1)	0.11
Monthly dosage of PSL just before IPAA (mg)	0(0–300)	0(0–300)	450(38–833)	0.0005
Biologic therapy use (Y/N)	52/263	49/246	3/17	1.00
Thiopurine use (Y/N)	56/259	53/242	3/17	1.00
Calcineurin Inhibitor use (Y/N)	34/281	31/264	3/17	0.46
Staged operation: two-stage/three-stage	207/108	191/104	16/4	0.22
Approach: Laparoscopy (Y/N)	130/185	128/167	2/18	0.004
Intraoperative blood infusion (Y/N)	25/290	22/273	3/17	0.20
Surgical site infection (Y/N)	28/287	26/269	2/18	0.69
Chronic pouchitis (Y/N)	72/243	59/236	13/7	<0.0001
Preceding chronic pouchitis (Y/N)	69/246	59/236	10/10	0.004
Follow-up period (months)	92(48–139)	93(49–142)	65(43–110)	0.06

IPAA = ileal pouch-anal anastomosis; Y = yes; N = no; PSL = prednisolone; FD = fistulous disease; + = positive; − = negative.

### Characteristics of patients with FD of the pouch

Table 1 summarizes the clinicopathological characteristics of the patients with and those without FD of the pouch. The age at UC onset and the age at surgery were significantly younger for the UC patients with FD than for those without FD of the pouch (26.5 ± 11.3 years vs. 33.1 ± 14.9 years, *p* = 0.04, and 31.3 ± 10.4 years vs. 40.5 ± 15.2 years, *p* = 0.007, respectively). The proportion of patients who underwent IPAA before June, 2010 was higher than that after June 2010 (60.0% vs. 34.2%, respectively; *p* = 0.03). The monthly PSL dosage just before IPAA was significantly higher for patients with than for those without FD of the pouch (494 ± 477 mg vs. 194 ± 33 5 mg, respectively; *p* = 0.0005). The proportion of patients who underwent laparoscopic surgery was significantly lower among patients with FD than among those without FD of the pouch (10.0% vs. 43.4%, respectively; *p* = 0.004). The proportion of patients with chronic pouchitis was significantly higher among those with FD than among those without FD of the pouch (65.0% vs. 20.0%, respectively; *p* < 0.0001). The proportion of patients with preceding chronic pouchitis was also significantly higher for those with FD than for those without FD of the pouch (50.0% vs. 20.0%, respectively; *p* = 0.004).

### Risk factors for FD of the pouch

To clarify the potential association of a preoperative medication as a risk predictor for FD of the pouch, we performed a Cox proportional hazards regression analysis for FD of the pouch-free survival (Table 2**)**. The results of the univariate analysis revealed that a monthly dosage of PSL ≥ 450 mg before IPAA was a significant risk factor for FD of the pouch, as were chronic pouchitis and preceding chronic pouchitis (*p* = 0.001, 0.0009, and 0.03, respectively). Since chronic pouchitis and preceding chronic pouchitis are confounding variables, we selected chronic pouchitis, which has a broader definition, as the covariate for the multivariable analysis. These factors were entered in the multivariate analysis, which identified both as independent risk factors for FD of the pouch (monthly PSL ≥ 450 mg before IPAA: odds ratio: 3.55, 95% confidence interval: 1.45–8.66, *p* = 0.006 and chronic pouchitis: odds ratio: 4.03, 95% confidence interval: 1.59–10.20, *p* = 0.003).

**Table 2:** Cox proportional hazards regression analysis for fistulizing disease of the pouch-free survival

Variable		Univariate			Multivariate		
		Odds ratio	95% CI	*p*-value	Odds ratio	95% CI	*p*-value
Sex	Female	1.59	0.66–3.84.66.84	0.30			
Age at ulcerative colitis onset	<18 years	1.19	0.40–3.57.40.57	0.75			
Age at IPAA	<18 years	1.76	0.41–7.59.41.59	0.45			
Year of IPAA	Before June 2010	1.77	0.71–4.44.71.44	0.22			
Disease duration	<10 years	2.30	0.67–7.85.67.85	0.18			
Extent of colitis	Total colitis	1.08	0.32–3.70.32.70	0.90			
Disease type	First attack	1.72	0.58–5.16.58.16	0.33			
Severity of colitis	Severe	1.14	0.15–8.50.15.50	0.90			
Cancer/dysplasia	Presence	0.41	0.055–3.08.055.08	0.39			
Pyoderma gangrenosum	Presence	1.65	0.22–12.31.22.31	0.63			
Body mass index	<19.4	2.37	0.94–5.94.94.94	0.07			
Monthly dosage of PSL before IPAA	≥450 mg	4.32	1.78–10.47.78.47	0.001	3.55	1.45–8.66.45.66	0.005
Biologic therapy use	Presence	1.22	0.36–4.21.36.21	0.75			
Thiopurine use	Presence	0.85	0.25–2.89.25.89	0.79			
Calcineurin Inhibitor use	Presence	1.54	0.45–5.26.45.26	0.49			
Staged operation	Two-stage	1.94	0.65–5.80.65.80	0.24			
Approach	Laparoscopy	0.24	0.055–1.05.055.05	0.06			
Intraoperative blood infusion	Presence	1.85	0.54–6.34.54.34	0.33			
Surgical site infection	Presence	0.87	0.20–3.78.20.78	0.85			
Chronic pouchitis	Presence	4.76	1.90–11.95.90.95	0.0009	4.03	1.59–10.20.59.20	0.003
Preceding chronic pouchitis	Presence	2.59	1.07–6.22.07.22	0.03			

IPAA = ileal pouch-anal anastomosis; PSL = prednisolone; FD = fistulous disease; CI = confidence interval.

### Risk impact of a high monthly dosage of PSL before IPAA and chronic pouchitis on the development of FD of the pouch

**Fig. 3:**
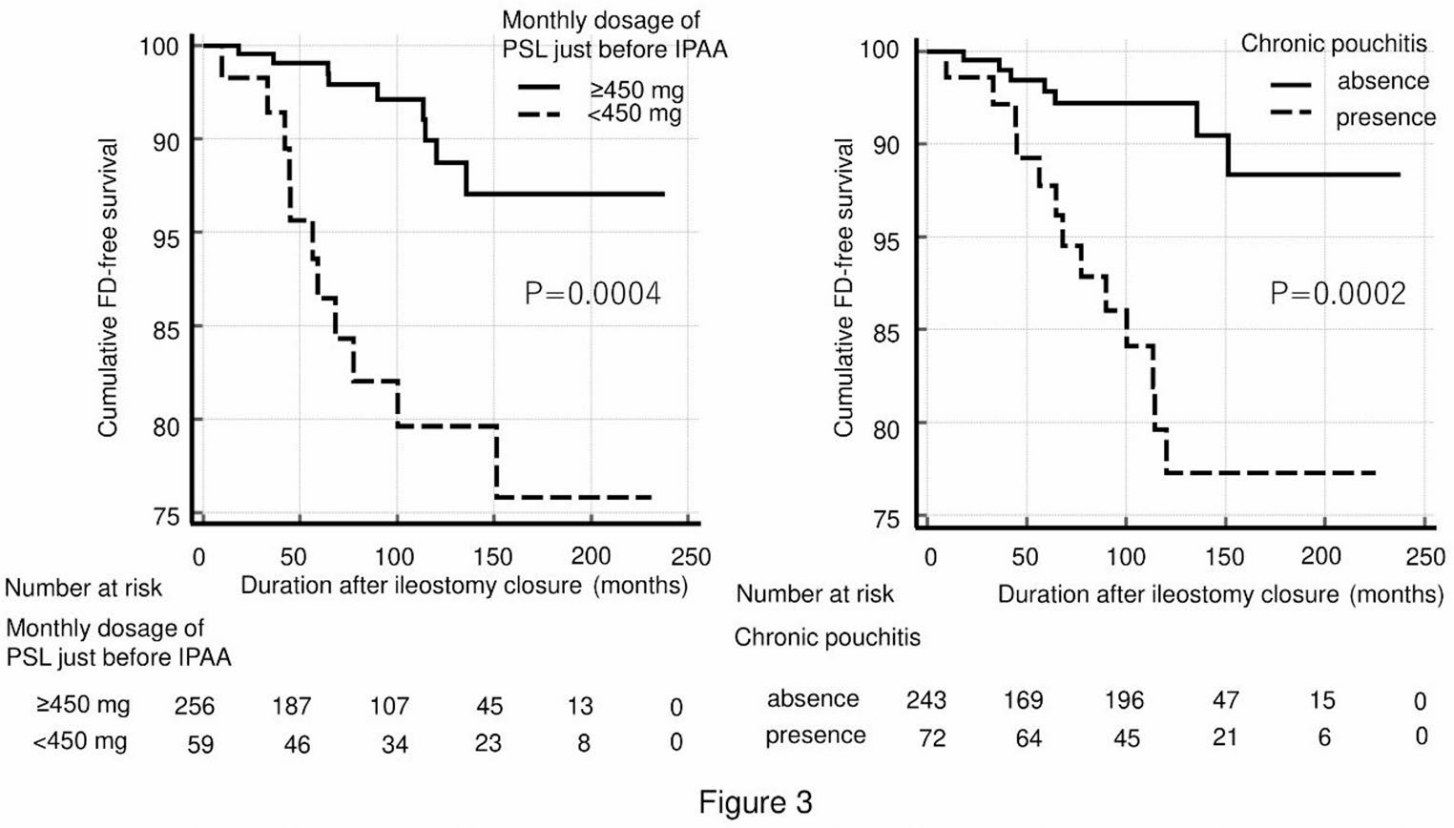
Kaplan–Meier survival curves subdivided by the monthly dosage of prednisolone (PSL) before ileal pouch-anal anastomosis (IPAA) and the presence of chronic pouchitis. Patients with a monthly dosage of PSL ≥ 450 mg before IPAA and chronic pouchitis had significantly poorer fistulizing disease of the pouch-free survival than those with a monthly dosage of PSL < 450 mg before IPAA and without chronic pouchitis (log-rank test, *p* = 0.0004 and *p* = 0.0002, respectively). IPAA = ileal pouch-anal anastomosis; PSL = prednisolone; FD = fistulizing disease

According to the Kaplan–Meier survival curves classified by the monthly dosage of PSL before IPAA and the presence of chronic pouchitis, patients with both factors had significantly poorer FD of the pouch-free survival than those with neither factor (log-rank test, *p* = 0.0004 and *p* = 0.0002, respectively; Fig. 3).

### Association between FD of the pouch and pouch failure

**Fig. 4:**
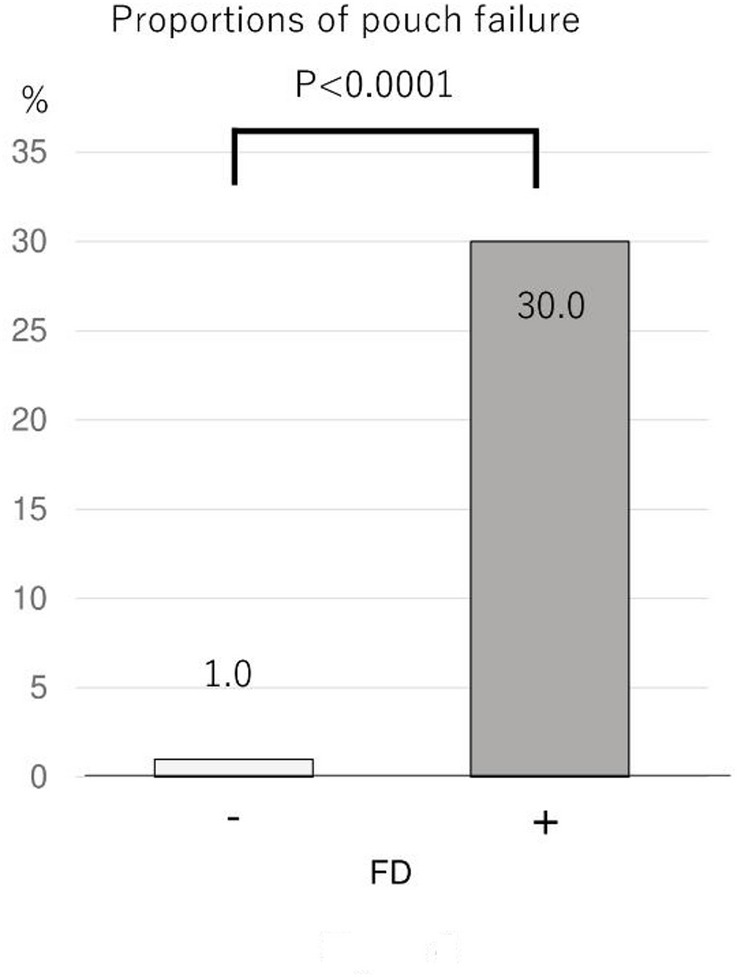
Comparison of the proportions of patients with pouch failure. The proportion of patients with pouch failure was significantly higher among those with fistulizing disease (FD) than among those without FD of the pouch (30.0% vs. 1.0%, respectively; *p* < 0.0001). FD = fistulizing disease

Pouch failure occurred in 9 (2.9%) of the 315 patients. The exact causes of pouch failure were uncontrollable FD of the pouch in six patients, anal sphincter dysfunction in two, and cancer recurrence in one. The proportion of patients who developed pouch failure was significantly higher among those with FD than among those without FD of the pouch (30.0% vs. 1.0%, respectively; *p* < 0.0001) (Fig. 4). Among the nine patients with pouch failure, two required pouch excision, and seven were left with a permanent diversion.

## Discussion

This study identified that FD of the pouch in UC patients with ileostomy closure after IPAA and chronic pouchitis was significantly associated with a high monthly dose of steroids just before IPAA. Furthermore, we found that FD of the pouch was the most common and significant cause of pouch failure.

FD of the pouch occurs after IPAA in 6%–37% of patients [18–21] and is associated with a high risk of pouch failure [22, 23]. One study reported that the overall incidence of postoperative perianal fistulas in patients with a preoperative diagnosis of UC, who underwent IPAA, was 9% [11]. Moreover, patients who were young at disease onset, comparatively younger at surgery, had indeterminate colitis, or were subsequently diagnosed with CD were more likely to develop perianal fistulas. Tekkis et al. [24] evaluated the risk factors for developing ileal pouch-related fistula following IPAA and found that a diagnosis of indeterminate colitis or CD, female sex, and pelvic sepsis were significantly associated with ileal pouch-related fistula.

A Japanese multicenter nationwide cohort study showed that the histopathologic diagnosis of UC was changed in 0.9% of the patients after surgery (0.7% patients with newly diagnosed CD and 0.2% with indeterminate colitis) [25]. In this study, CD was newly diagnosed postoperatively in only two patients. The incidence of newly diagnosed CD following surgery in Japan could be lower than that in previous reports [26–29] from Western countries. Generally, a fistula without a history of pouch leakage and/or the late development of fistulas or a sinus after ileostomy closure in UC patients should raise suspicion of CD of the pouch [30, 31]. However, the diagnosis of CD of the pouch in UC patients remains challenging because of the inconsistency in defining CD of the pouch and in the terminology used to describe an inflammatory Crohn’s-like condition in patients with IPAA [4, 15]. Recent studies have found that CD of the pouch can develop in a subset of patients with UC followed by IPAA and is the most common reason for pouch failure. A meta-analysis identified that CD of the pouch was diagnosed in 10.3% of 4843 patients with an IPAA for UC or indeterminate colitis [4]. Sossenheimer et al. [32]. suggested that CD of the pouch might be over-diagnosed because all patients with extravasation leading to pouch failure were eventually considered to have CD based on clinical findings rather than strictly on histopathologic criteria. Reijntjes et al. [7]. also reported that a misdiagnosis of CD of the pouch in patients with long-term surgical sequelae could lead to overtreatment with immunosuppressive therapies, whereas alternative treatments, such as surgical intervention, might be indicated. These findings prompt consideration of an examination of the risks of FD of the pouch, excluding patients diagnosed with CD.

To our knowledge, this is the only study to identify that a high dose of steroids just before surgery is an independent risk factor for FD of the pouch long-term in UC patients. Previous studies have reported an association between a high cumulative preoperative PSL dose and short-term outcomes after IPAA [33, 34]. The results showed that a high cumulative preoperative PSL dose was an independent risk factor for postoperative infectious complications within 30 days [33, 34]. Notably, regarding the long-term effects of PSL, the effects on adaptive immunity, including T-lymphocyte apoptosis, may be long-lasting and can result in failure to generate pathogen-specific adaptive immune responses [35]. Moreover, the trophic effects of glucocorticoids on connective tissue may weaken barrier function and result in long-term changes in host vulnerability to infection [36, 37]. It is likely that microleakage or infections occur at the anastomotic site of the ileal pouch mucosa or the anal canal mucosa, and that some time passes before the development of clinically-apparent FD.

In this study, we observed a significant association between a monthly PSL dose ≥ 450 mg prior to IPAA and severe disease (PSL dose ≥ 450 mg: severe 11.9% vs. PSL dose < 450 mg: severe 2.7%, *p* = 0.007). Patients on excessive steroid doses may reflect greater disease severity, and latent disease activity could contribute to the development of FD. However, the monthly PSL dose does not necessarily correspond to the period before the initial surgery. In three-stage procedures, it represents the period before the second surgery; therefore, steroid overdose does not always directly indicate disease severity at the time of IPAA.

Multiple studies indicate that preoperative biologic therapy does not increase the risk of anastomotic leakage or other postoperative complications significantly in patients with UC undergoing surgery [38–40]. Although the number of patients who received biologic therapy before IPAA in this study was limited, biologic use before IPAA may not increase the risk of FD of the pouch.

Shen et al. proposed further stratification of patients with CD of the pouch into three main categories based on their main phenotype [26]. These categories are inflammatory, fibrostenotic, and fistulizing CD of the pouch, which is the most common cause of pouch failure. Reza et al. [41] proposed that pouch-anal fistulas should be classified into four distinct groups according to their etiology. Group 2 (type B) fistulas are a distinct subgroup not associated with features consistent with CD, but with the histological characteristics of chronic inflammation in the pouch or cuff. van der Ploeg [42] et al. reported that nearly 40% of patients thought to have chronic pouchitis had chronic peripouch sepsis. Concurrent chronic pouchitis and FD of the pouch is not uncommon. In our study, chronic pouchitis was identified as an independent risk factor for FD of the pouch. This finding suggests that FD is attributed to inflammation specific to the pouch, and FD of the pouch caused by chronic pouchitis could be classified as group 2 (type B) in accordance with the definition proposed by Reza et al. Although inflammation of the rectal cuff has been suggested to contribute to fistula formation in stapled IPAA [41], the present study analyzed only patients who underwent handsewn IPAA. Thus, it is possible that chronic pouchitis contributed to fistula formation even in handsewn IPAA pouches. Persistent inflammation of the pouch distinct from CD of the pouch may cause fistulas or FD of the pouch. The prevention or appropriate treatment of chronic pouchitis might reduce the incidence of FD of the pouch.

This study had several limitations, including its retrospective design and the small number of patients. Moreover, the data period spanned nearly 20 years, during which time the surgical procedure evolved. Although FD was significantly more frequent before June 2010, a univariate analysis using the Cox proportional hazards model did not demonstrate a significant difference in FD of the pouch-free survival. Furthermore, we identified a tendency toward different follow-up durations between patients with FD and those without FD of the pouch, although the difference was not significant.

## Conclusions

This study identified that FD of the pouch in UC patients with ileostomy closure after IPAA was significantly associated with a high monthly dose of steroids just before IPAA and chronic pouchitis. Based on these results, we hypothesize that FD of the pouch may be associated with tissue vulnerability caused by steroids or persistent inflammation of the pouch. Understanding that FD of the pouch may involve different pathological mechanisms than those associated with CD of the pouch could help avoid overtreatment of patients on immunosuppressive therapies.

## Supplementary Information

Below is the link to the electronic supplementary material.

Supplementary material 1 (DOCX 28.3 kb)
