# Supplementary material for: Risk factors for fistulizing disease of the pouch after ileal pouch-Anal anastomosis for ulcerative colitis
Source: Surg Today. 2026 Feb 9;56(7):1360–8. doi: 10.1007/s00595-026-03246-8 (PMC13303665; doi:10.1007/s00595-026-03246-8)
Supplement: Supplementary file 1 — Supplementary material 1 (DOCX 28.3 kb) [file 595_2026_3246_MOESM1_ESM.docx]

**Supplemental Table 1.** Procedures used to identify fistulizing disease of the pouch and exclude Crohn’s disease, and treatments for fistulizing disease of the pouch

| Patients | Age at IPAA  (years) | Sex | FD | Diagnostic procedures | | | | Treatments |
| --- | --- | --- | --- | --- | --- | --- | --- | --- |
|  |  |  |  | PS | CT | GE | MRI |  |
| 1 | 24 | M | PPF | Y | Y | Y | N | Seton drainage, IFX |
| 2 | 50 | F | PPF | Y | Y | Y | N | Seton drainage, Permanent ileostomy |
| 3 | 29 | F | PVF | Y | Y | Y | Y | Temporary ileostomy |
| 4 | 29 | F | ELL | Y | Y | Y | N | Closure of the efferent limb leakage |
| 5 | 28 | M | ELL | Y | Y | Y | Y | Permanent ileostomy |
| 6 | 33 | F | PPF, ELL | Y | Y | Y | N | Seton drainage, Permanent ileostomy |
| 7 | 32 | M | ELL | Y | Y | Y | N | Closure of the efferent limb leakage, Temporary ileostomy |
| 8 | 22 | M | PSS | Y | Y | Y | N | Temporary ileostomy |
| 9 | 44 | F | PVF , PPF | Y | Y | Y | N | Seton drainage, Permanent ileostomy |
| 10 | 17 | M | PBL, PSS, PPF | Y | Y | Y | N | Seton drainage, Permanent ileostomy |
| 11 | 17 | F | PPF | Y | N | Y | N | Seton drainage |
| 12 | 32 | M | ELL | Y | Y | Y | Y | Closure of the efferent limb leakage, Temporary ileostomy |
| 13 | 32 | M | PPF | Y | Y | Y | N | Incision with drainage |
| 14 | 21 | M | PPF | Y | Y | Y | Y | Seton drainage |
| 15 | 29 | F | PVF | Y | Y | Y | Y | No treatment |
| 16 | 31 | F | PVF | Y | Y | Y | Y | No treatment |
| 17 | 47 | F | PVF | Y | Y | Y | N | Permanent Ileostomy |
| 18 | 30 | F | PPF | Y | Y | Y | N | Seton drainage |
| 19 | 55 | M | PSS | Y | Y | Y | N | No treatment |
| 20 | 25 | F | PPF | Y | Y | Y | Y | Temporary Ileostomy |

IPAA = ileal pouch-anal anastomosis; FD = fistulizing disease; PS = pouchoscopy; GE = gastrographin enema; MRI = magnetic resonance imaging; M = male; F = female; PPF = pouch-perianal fistula; PVF = pouch-vaginal fistula; ELL = efferent limb leakage; PSS = presacral sinus; PBL = pouch-body leakage; IFX = infliximab; Y = yes; N = no.
